# Lesioned hemisphere‐specific phenotypes of post‐stroke fatigue emerge from motor and mood characteristics in chronic stroke

**DOI:** 10.1111/ene.16170

**Published:** 2023-12-09

**Authors:** William De Doncker, Annapoorna Kuppuswamy

**Affiliations:** ^1^ Department of Clinical and Movement Neuroscience, Institute of Neurology University College London London UK; ^2^ Department of Biomedical Sciences University of Leeds Leeds UK

**Keywords:** k‐means clustering, motor cortex excitability, phenotypes, post‐stroke fatigue

## Abstract

**Background and purpose:**

Post‐stroke fatigue commonly presents alongside several comorbidities. The interaction between comorbidities and their relationship to fatigue is not known. In this study, we focus on physical and mood comorbidities, alongside lesion characteristics. We predict the emergence of distinct fatigue phenotypes with distinguishable physical and mood characteristics.

**Methods:**

In this cross‐sectional observational study, in 94 first time, non‐depressed, moderate to minimally impaired chronic stroke survivors, the relationship between measures of motor function (grip strength, nine‐hole peg test time), motor cortical excitability (resting motor threshold), Hospital Anxiety and Depression Scale and Fatigue Severity Scale‐7 (FSS‐7) scores, age, gender and side of stroke was established using Spearman's rank correlation. Mood and motor variables were then entered into a k‐means clustering algorithm to identify the number of unique clusters, if any. Post hoc pairwise comparisons followed by corrections for multiple comparisons were performed to characterize differences among clusters in the variables included in k‐means clustering.

**Results:**

Clustering analysis revealed a four‐cluster model to be the best model (average silhouette score of 0.311). There was no significant difference in FSS‐7 scores among the four high‐fatigue clusters. Two clusters consisted of only left‐hemisphere strokes, and the remaining two were exclusively right‐hemisphere strokes. Factors that differentiated hemisphere‐specific clusters were the level of depressive symptoms and anxiety. Motor characteristics distinguished the low‐depressive left‐hemisphere from the right‐hemisphere clusters.

**Conclusion:**

The significant differences in side of stroke and the differential relationship between mood and motor function in the four clusters reveal the heterogenous nature of post‐stroke fatigue, which is amenable to categorization. Such categorization is critical to an understanding of the interactions between post‐stroke fatigue and its presenting comorbid deficits, with significant implications for the development of context‐/category‐specific interventions.

## INTRODUCTION

Post‐stroke fatigue is a significant and highly prevalent symptom in chronic stroke [1, 2, 3]. Although commonly seen alongside other post‐stroke disturbances such as depression, anxiety, cognitive dysfunction and motor impairment, fatigue is an independent symptom and is poorly understood [4, 5, 6, 7, 8].

An extensive body of literature is dedicated to studying the relationship between fatigue, lesion location, impairment and mood disorders [6, 9, 10, 11, 12, 13]. While fatigue incidence is greater when the lesion includes the thalamus, when there is significant depression and anxiety, and sometimes in the presence of physical and cognitive impairment, it is still unclear how these different factors interact and, in combination, explain fatigue incidence and severity. For example, in some chronic stroke survivors, fatigue can emerge in the absence of physical, cognitive or mood deficits, while in others it occurs in the presence of one or more deficits. It is critically important that we identify the contexts, that is, the combination of factors that co‐present with fatigue, and establish if there are certain contexts that are more conducive to fatigue. The need for such understanding is highlighted in intervention studies which show that responsiveness to a treatment is dependent on the context (phenotype) of fatigue [14]. In this study we aimed to identify fatigue phenotypes, based on factors related to post‐stroke fatigue.

Low motor cortical excitability of the stroke lesioned hemisphere is seen in high post‐stroke fatigue [15], and was included as the neural correlate of fatigue for phenotyping. While side of lesion is not associated with either incidence or severity of fatigue, balance of interhemispheric inhibition is reversed, with a right hemispheric dominance associated with high fatigue [16]. This shift in interhemispheric inhibition is independent of side of stroke, indicating that shift in laterality is not related to stroke lesion, but to fatigue. The shift in laterality of inhibition is likely to influence motor cortical excitability of the lesioned hemisphere, however its impact is not known. Therefore, side of stroke lesion, motor cortical excitability, along with measures of hand motor function were included in this study as physical factors in phenotyping fatigue.

Post‐stroke fatigue is correlated with depression and anxiety [10], therefore, measures of mood were included in phenotyping of fatigue. There is a known effect of stroke side on depression [17], with left‐hemisphere strokes carrying a greater burden of depression. Given the impact of side of stroke on both motor physiology and mood, to avoid large side‐related effects on depression overshadowing its effect on motor physiology, we excluded those with clinically diagnosed depression.

To summarize, in this investigation, we first identified the association between post‐stroke fatigue, motor function, motor physiology, depressive symptoms and anxiety, then categorized high fatigue into clusters to define the motor and mood characteristics of each category. We expected to replicate previously observed associations of post‐stroke fatigue, with an emergence of distinguishable clusters driven by motor, mood, and side of stroke.

## MATERIALS AND METHODS

### Subjects

This cross‐sectional observational study was approved by the London Bromley Research Ethics Committee (REC reference number: 16/LO/0714). A total of 94 stroke survivors who had experienced first‐time stroke and were ≥3 months post‐stroke were included in the study. Exclusion criteria were: (i) diagnosis of any other neurological disorder; (ii) receiving treatment with anti‐depressants or centrally acting drugs; (iii) depression scores ≥11 assessed using the Hospital Anxiety and Depression Scale (HADS); (iv) grip strength or manual dexterity (nine‐hole peg test [NHPT] time) of the affected hand being <60% of the unaffected hand; and (v) contraindications to transcranial magnetic stimulation (TMS). Table 1 shows the demographic characteristics of the 94 stroke survivors). All stroke survivors provided written informed consent in accordance with the Declaration of Helsinki.

**TABLE 1:** Demographics of patients who completed the study. Mean (SD) values are displayed for all continuous variables and number of patients are displayed for categorical variables (hemisphere affected, gender, dominant hand).

	All patients (*N* = 94)	Left hemisphere stroke (*N* = 51)	Right hemisphere stroke (*N* = 47)	*p* value
FSS‐7 score, mean (SD)	3.83 (1.91)	3.59 (1.94)	4.09 (1.85)	
Hemisphere affected				
Left | Right	51 | 47			0.20
Gender				
Female | Male	32 | 66	21 | 30	11 | 36	0.003; 0.002; 0.200
Dominant hand				
Right | Left	88 | 10	47 | 4	41 | 6	0.128; 0.765; 0.064
Age, years	60.93 (12.65)	59.69 (13.40)	62.28 (11.78)	0.050; 0.008; 0.911
Grip strength, % unaffected hand	91.37 (22.93)	96.96 (19.14)	85.31 (25.27)	0.044; 0.454; 0.130
NHPT time, % unaffected hand	88.48 (24.63)	97.98 (22.46)	78.16 (22.85)	0.226; 0.689; 0.212
HADS‐Depression score	4.83 (3.23)	4.29 (3.11)	5.40 (3.30)	<0.001; <0.001; 0.002
HADS‐Anxiety score	5.26 (3.93)	4.71 (4.25)	5.85 (3.49)	<0.001; 0.0084; 0.0162
RMT for affected hemisphere, % MSO	47.43 (11.90)	48.47 (11.88)	46.30 (11.94)	0.791; 0.210; 0.416

*Note*: *p* values indicate the significance of spearman rank correlations between Fatigue Severity Scale‐7 (FSS‐7) score and continuous variables and Wilcoxon rank sum tests comparing the FSS‐7 score within categorical values for the entire patient cohort and the two groups divided based on hemisphere affected by the stroke.

Abbreviations: HADS, Hospital Anxiety and Depression Scale; MSO, maximum stimulator output; NHPT, nine‐hole peg test; RMT, resting motor threshold.

### Fatigue quantification

Fatigue was measured using the Fatigue Severity Scale‐7 (FSS‐7), a widely used and validated questionnaire to measure self‐reported fatigue across different conditions including stroke [18]. An average score of 4 out of a maximum of 7 was used as the lower limit for defining the ‘high fatigue’ group, as is commonly applied in clinical practice.

### Mood quantification

The validated and widely used HADS questionnaire was used to measure both anxiety and depressive symptoms. A maximum score of 21 can be obtained for each of the Anxiety and Depression subsections. A score of 11 or below is considered to be in the non‐depressive range, although the higher the score, the greater the likelihood of depression‐like symptoms. While we excluded anyone with a depression subsection score greater than 11, there was no exclusion based on anxiety scores.

### Motor function

Hand grip strength was measured using a hand‐held dynamometer. Three repetitions of maximum voluntary grip were performed on each hand in randomized order with adequate rest between contractions, with the average value from three contractions taken as the maximum voluntary force for a given hand. Grip function was calculated as follows:

$$\text{Grip}=\text{average grip}\;\left[\left(\text{affected hand}\right)/\text{average grip}\;\left(\text{unaffected hand}\right)\right]\times 100.$$

The average time taken to complete three repetitions of the NHPT was taken as the dexterity score. Dexterity function was calculated as follows:

$$\text{Dexterity}=\text{average dexterity score}\;\left[\left(\text{affected hand}\right)/\text{average dexterity score}\;\left(\text{unaffected hand}\right)\right]\times 100.$$

### Motor physiology: Surface electromyogram and transcranial magnetic stimulation

Electromyogram recordings were obtained from the first dorsal interosseous (FDI) muscle using surface neonatal prewired disposable electrodes (1041PTS Neonatal Electrode, Kendell) in a belly‐tendon montage with the ground positioned over the flexor retinaculum of the hand. The signal was amplified with a gain of 1000 (D360, Digitmer), bandpass filtered (100–1000 Hz), digitized at 5 kHz (Power1401, CED) and recorded with Signal version 6.04 software (CED).

A TMS device (Magstim 200^2^, Magstim) connected to a figure‐of‐eight coil (wing diameter, 70 mm) was used to stimulate the hand area of M1 in each hemisphere. The coil was held tangentially on the scalp at an angle of 45° to the mid‐sagittal plane to induce a posterior–anterior current across the central sulcus. The subjects were instructed to stay relaxed with their eyes open and their legs uncrossed. The motor ‘hotspot’ of the FDI muscle for each hemisphere was determined as previously [15].

The resting motor threshold (RMT) for each hemisphere was defined as the lowest intensity of stimulation (% maximum stimulator output) required to evoke a peak‐to‐peak motor evoked potential amplitude at the hotspot of at least 50 μV in a minimum of five out of 10 consecutive trials while the subjects were at rest.

### Statistical analysis

Spearman's rank correlations between FSS‐7 score and age, anxiety, depression, grip strength, NHPT time, symbol digits modalities test (SDMT) and RMT were calculated. Wilcoxon rank‐sum tests were used to assess the difference in FSS‐7 scores across different groups divided according to gender, the hemisphere affected, and the dominant hand affected.

Multiple linear regression analysis was used to examine the effect of RMT on FSS‐7 score in all patients and in two groups based on the hemisphere affected by the stroke. Assumptions of normality and homoscedasticity of the residuals for each linear regression model were assessed visually using quantile‐quantile normal plots and fitted versus residual value plots. The level of significance was set at *p* = 0.05.

### k‐means clustering analysis

Of the 94 patients included in the regression analyses, 47 had an FSS‐7 score of ≥4. This subset of 47 patients was defined as the high fatigue group and were subject to k‐means clustering analysis. The motor variables grip, NHPT time and RMT were compressed into a single variable using principal component analysis (PCA). The average of the first two principal components was used as the motor PCA score. The first component almost exclusively represented data from functional measures and the second principal component represented data from RMT, hence the average of the first two components was taken to represent the overall motor score. *Z*‐scores were calculated for the four variables of interest (HADS‐Anxiety, HADS‐Depression, stroke side and motor PCA score) and were entered into the k‐means clustering algorithm. A k‐means cluster analysis is an unsupervised machine‐learning algorithm‐based technique used to identify subgroups (or clusters) in a dataset that represent data points that are similar to one another, yet distinct from data points in other clusters. The *k*‐means algorithm clusters the data into a number, *k*, of predefined, distinct, and non‐overlapping groups where each data point only belongs to one group. Data points are assigned to a particular cluster in such a way that the sum of the squared distance between all of the data points, and the mean of all the data points that belong to that cluster, is minimized. Based on known associations between post‐stroke fatigue, motor function, motor physiology and mood characteristics, we tested k values of 2–5. The best model was chosen from silhouette scores, a score that indicates robustness of clustering. A score of 1 indicates completely non‐overlapping clusters, while a score of 0 means there is no difference between clusters, and a score of −1 indicates wrong classification. A score between 0.3 and 0.7 is normally considered acceptable for identifying unique non‐overlapping clusters. The model with the highest silhouette score above 0.3 was considered the winning model in this study, and the number of clusters was determined from this. Given the non‐parametric nature of the data, a Kruskal–Wallis analysis of variance (ANOVA) test, followed by post hoc comparisons using Dunn's method adjusted for multiple comparisons using Bonferroni correction, were performed to identify differences between the various clusters in the parameters of interest.

## RESULTS

There was a significant association between FSS‐7 score and grip strength (*ρ* = −0.204, *p* = 0.044), HADS‐Anxiety score (*ρ* = 0.37, *p* < 0.001) and HADS‐Depression score (*ρ* = 0.50, *p* < 0.001) in 94 patients. When patients were divided into groups based on hemisphere affected, there was a significant association between FSS‐7 score and age (*ρ* = −0.37, *p* = 0.008), anxiety (*ρ* = 0.37, *p* = 0.008) and depression (*ρ* = 0.53, *p* < 0.001) in left‐hemisphere strokes and a significant positive association between FSS‐7 score and HADS‐Anxiety (*ρ* = 0.35, *p* = 0.016) and HADS‐Depression scores (*ρ* = 0.44, *p* = 0.002) in right‐hemisphere strokes. There was a significant difference in median FSS‐7 score between men and women in the entire cohort and in those with left‐hemisphere strokes (*p* = 0.003; *p* = 0.002). There was no association between FSS‐7 score, action research arm test (ARAT) and RMT of the affected hemisphere and no difference in FSS‐7 score in left‐hemisphere strokes compared to right‐hemisphere strokes. Patient demographics can be found in Table 1.

**FIGURE 1:**
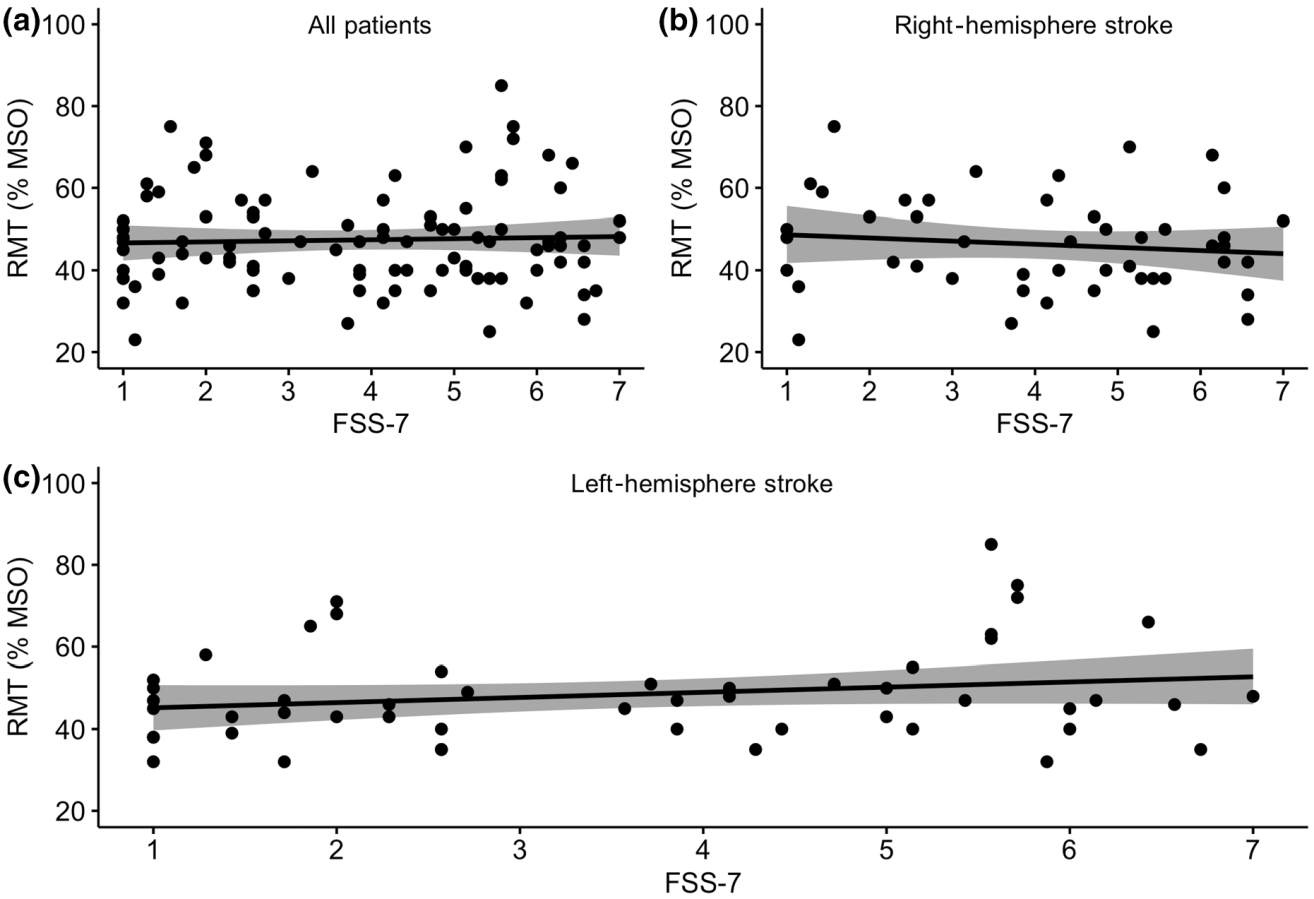
Regression line with 95% confidence interval and individual data points showing the association between resting motor threshold (RMT) and self‐reported fatigue (Fatigue Severity Scale‐7 [FSS‐7]) in the entire cohort of stroke patients (a), patients with right‐hemisphere strokes (b) and patients with left‐hemisphere strokes (c). FSS‐7 score was a significant predictor of RMT in patients with left‐hemisphere strokes. MSO, maximum stimulator output.

The best‐fit regression model that predicted FSS‐7 score in the entire sample included RMT, HADS‐Anxiety score and age (*F*_3,94_ = 5.97, *p* < 0.001), with an adjusted *R* ^2^ of 0.133 (Figure 1a). When patients were divided into two groups based on stroke hemisphere, in left‐hemisphere stroke, the combined predictive power of RMT (*β* = 0.047, *p* = 0.025 [confidence interval {CI} 0.006, 0.090]), HADS‐Anxiety (*β* = 0.162, *p* = 0.008 [CI 0.044, 0.280]) and age (*β* = −0.044, *p* = 0.017 [CI −0.081, −0.008]) explained FSS‐7 scores (*F*_3,47_ = 6.38, *p* = 0.001), with an adjusted *R* ^2^ of 0.244 (Figure 1c). There was no significant FSS‐7 predictor for right‐hemisphere strokes (*F*_3,43_ = 2.16, *p* = 0.106; Figure 1b).

**FIGURE 2:**
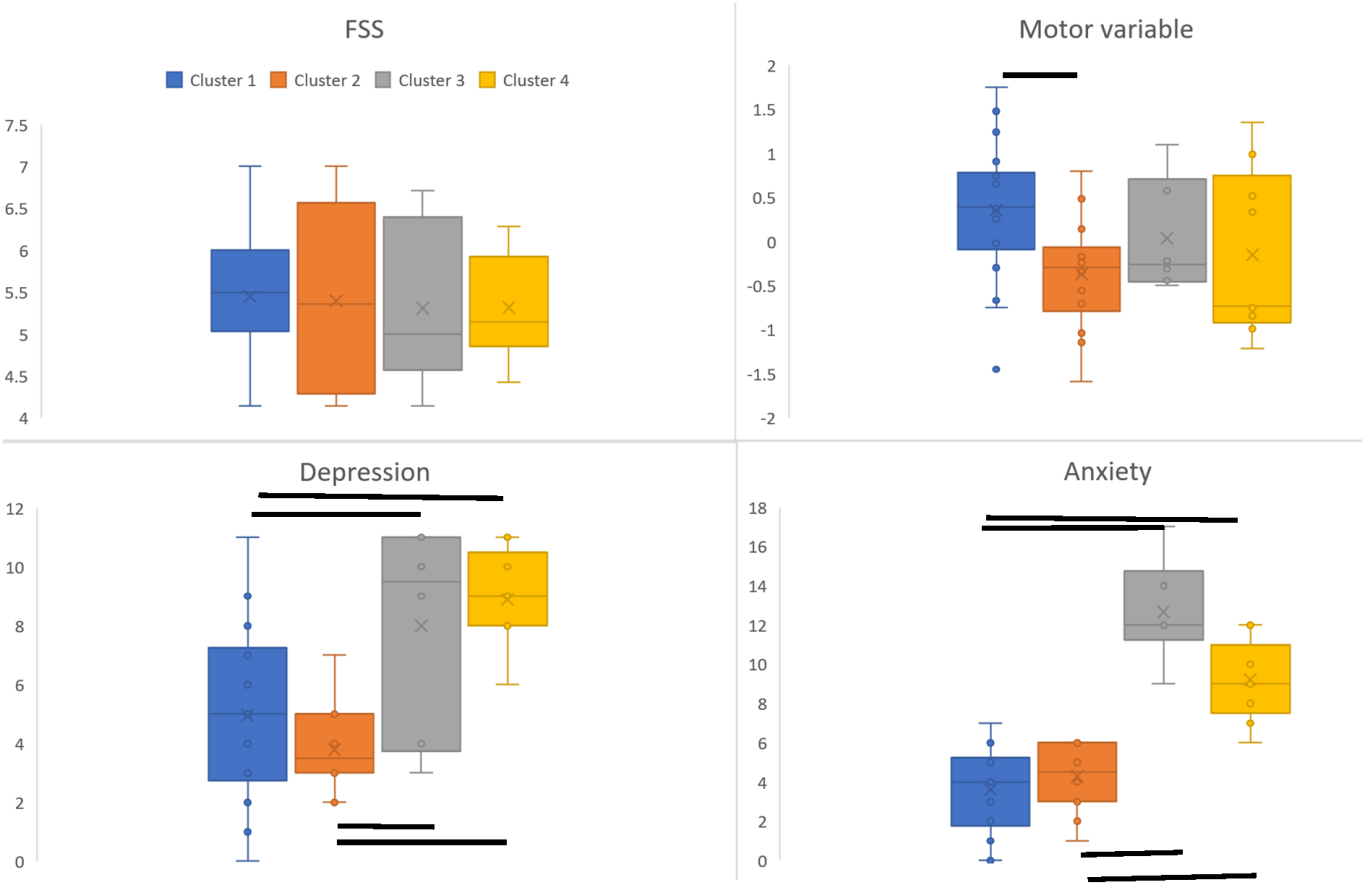
Box and whisker plots showing differences between clusters in the motor variable (motor PCA score), depressive symptoms and anxiety. Clusters 1 and 3 included left‐hemisphere strokes while Clusters 2 and 4 included right‐hemispheric strokes. Black lines represent significant differences between clusters.

Results of k‐mean cluster analysis indicated that a four‐cluster solution produced the best‐fit model, based on the highest silhouette score (0.311). The other models with two, three and five clusters had silhouette scores of 0.283, 0.287 and 0.258, respectively. The number of objects in each cluster was 18, 14, 6 and 9. Object‐wise silhouette scores, distance from centroid and correlation with centroid are provided in the supplementary methods. Cluster‐wise data are shown in Table 2. Kruskal–Wallis ANOVA was significant for three of the four factors tested (side of stroke *H* statistic = 46, *p* < 0.0001; HADS‐Depression *H* = 18.02, *p* < 0.0001; HADS‐Anxiety *H* = 30.048, *p* < 0.0001). Clusters 1 and 3 consisted of individuals with only left‐hemisphere stroke, and Clusters 2 and 4 consisted of only right‐hemisphere strokes. Post hoc pairwise comparisons showed a significant difference between Clusters 1 and 3 (*p* < 0.001), 1 and 4 (*p* = 0), 2 and 3 (*p* = 0) and 2 and 4 (*p* = 0.002) in both depression and anxiety scores, all of which survived Bonferroni correction. Motor PCA score was different between Clusters 1 and 2 (*p* = 0.015), which did not survive Bonferroni correction for multiple comparisons. Figure 2 shows cluster‐wise motor, HADS‐Depression, HADS‐Anxiety and FSS‐7 scores.

**TABLE 2:** Demographics of patients in each cluster. Mean (SD) values are displayed for all continuous variables and number of patients are displayed for categorical variables (hemisphere affected, gender, dominant hand).

	Cluster 1 (*n* = 18)	Cluster 2 (*n* = 14)	Cluster 3 (*n* = 6)	Cluster 4 (*n* = 9)	*p* value
FSS‐7 score, mean (SD)	5.45 (0.73)	5.39 (1.00)	5.3 (0.89)	5.31 (0.59)	NS
Hemisphere affected					
Left | Right	18 | 0	0 |14	6 | 0	0 | 9	**0.000** (1 and 2; 2 and 3; 1 and 4; 3 and 4)
Gender					
Female | Male	7 | 11	5 | 9	4 | 2	6 | 3	NS
Age, years	61.27 (11.53)	57.07 (12.27)	62.33 (16.66)	54.44 (11.74)	NS
Grip, % unaffected hand	91.82 (24.79)	84.66 (17.24)	93.98 (9.82)	94.3 (37.05)	NS
NHPT, % unaffected hand	99.28 (29.52)	76.54 (21.62)	91.66 (22.04)	85.39 (18.54)	NS
HADS‐Depression	4.94 (2.89)	3.78 (1.31)	8.0 (3.26)	8.88 (1.52)	**<0.001** (1 and 3, 1 and 4, 2 and 3, 2 and 4)
HADS‐Anxiety	3.61 (2.05)	4.28 (1.62)	12.66 (2.42)	9.22 (1.93)	**<0.001** (1 and 3, 1 and 4, 2 and 3, 2 and 4)
RMT for affected hemisphere, % MSO	52.15 (13.64)	43 (11.02)	43.5 (7.58)	47.33 (10.42)	NS
Motor PCA score_grip strength, NHPT, RMT_	0.35 (0.78)	−0.37 (0.6)	0.03 (0.59)	−0.14 (0.89)	0.015 (1 and 2)

*Note*: *p* values indicate the significance of Dunn's post hoc comparison, with cluster numbers within parenthesis showing to which clusters the *p* value corresponds. The numbers in bold are the significant *p* values.

Abbreviations: FSS‐7, Fatigue Severity Scale‐7; HADS, Hospital Anxiety and Depression Scale; MSO, maximum stimulator output; NHPT, nine‐hole peg test; PCA, principal component analysis; RMT, resting motor threshold.

## DISCUSSION

The main finding of this study was the emergence of four distinct phenotypes within high fatigue chronic stroke survivors, using an unsupervised clustering method that included variables of motor behaviour, motor neurophysiology, side of stroke and scores for depression and anxiety. Of the four clusters, two were composed only of left‐hemisphere stroke and two only of right‐hemisphere stroke. In left‐hemisphere strokes, the two clusters were differentiated by high and low levels of depressive symptoms and anxiety. Similarly, in right‐hemisphere strokes, levels of depressive symptoms and anxiety differentiated the two groups. There was a difference between left‐ and right‐hemisphere non‐depressive, non‐anxious clusters, in the motor variable. While gender differences across the clusters did not reach significance, there were more men in the non‐depressive, non‐anxious clusters and more women in the more anxious depressive clusters. In the entire sample of chronic stroke survivors, there was a significant positive relationship between fatigue levels and motor function, depression and anxiety, with only left‐hemisphere stroke displaying a positive correlation between fatigue and corticospinal excitability (CSE).

The emergence of four distinguishable clusters in mild to moderately impaired chronic stroke survivors shows, for the first time, that it is possible to categorize high fatigue into smaller non‐overlapping groups. In other disorders which present with complex behavioural profiles, such as autism spectrum disorder [19] and psychosis [20], phenotyping has been useful in identifying the underlying pathophysiology and in developing phenotype‐specific interventions. Here, we propose that phenotyping post‐stroke fatigue serves a similar purpose by helping define the characteristics of high fatigue groups, and this is in line with recommendations for post‐stroke fatigue research [21].

The difference in hemisphere affected, accompanied by differences in function and mood in the different clusters of high fatigue, is a novel finding of this study. The relationship between post‐stroke fatigue, impairment and mood have been previously investigated, however, the results have been conflicting, with some showing no relationship between fatigue and impairment [22, 23] and others revealing more nuanced interactions; for example, controlling for gender allowed significant relationships with impairment and mood to emerge [10]. In some studies where impairment was not included, mood appeared to be the main driver of post‐stroke fatigue [5]. While most previous investigations have included stroke hemisphere as a factor of interest, they all failed to reveal a role of stroke side in fatigue, possibly because patients with strokes of either hemisphere are equally likely to develop high fatigue, as shown by equal overall numbers of high fatigue in both right‐ and left‐hemisphere strokes in this study. However, what has been revealed here are the factors essential for side of stroke to influence development of fatigue in an individual. Several examples of how the current data might explain discrepancies in the literature can be elaborated, but larger studies with more variables are necessary before definitive conclusions about high fatigue clusters can be drawn. High levels of depressive symptoms and anxiety appear to be equally present in both right‐ and left‐hemisphere strokes, although only few individuals in both categories present this profile.

No clear differences were observed in motor thresholds that could enable us to distinguish left‐ and right‐hemisphere strokes with high fatigue, despite the overall sample showing a positive relationship between threshold and fatigue level in left‐hemisphere strokes. CSE assessed using TMS thresholds is highly variable in the general population [24], which was evident from the threshold values of the entire cohort in the present study. CSE of the left and right hemispheres behaves differently during motor control, with asymmetries in CSE seen between the two hemispheres [25, 26, 27]. The left hemisphere plays a dominant role in motor control, which is characterized by higher CSE when compared to the right hemisphere, broader activation patterns during movement, and greater thickness of the motor cortex [26, 28, 29]. The asymmetry in CSE between the two hemispheres is driven by inter‐hemispheric connectivity. In a normal brain, inter‐hemispheric connectivity is characterized by a net left‐to‐right inhibitory dominance and right‐to‐left excitatory dominance within the motor cortices and outside primary motor areas [30, 31, 32, 33], resulting in higher CSE of the left hemisphere when compared to the right. In post‐stroke fatigue, opposite patterns of inter‐hemispheric connectivity in primary motor cortices is seen, with a shift from net left‐to‐right to a net right‐to‐left inhibitory dominance [34]. Given the shift in inter‐hemispheric inhibition balance, those with high fatigue and left‐hemisphere stroke will present with lower excitability than those without fatigue. The results of the present study are in line with this expectation, however, the opposite is expected in right‐hemisphere strokes, that is, higher excitability is associated with high fatigue. Despite a trend, this did not reach significance in the present study.

### Potential applications and future directions

The identification of clusters in this investigation has the potential to help in the clinical management of post‐stroke fatigue and the development of symptom‐specific interventions. Clustering indicates that high fatigue manifests both in the presence and absence of high levels of anxiety. With previous evidence suggesting anxiety is a significant predictor of response to fatigue‐reducing interventions [35], any management strategy or fatigue intervention must aim to first reduce anxiety before attempting to manage fatigue. Furthermore, the likelihood of high fatigue in left‐hemisphere strokes, more so than right‐hemisphere strokes, irrespective of anxiety level, is associated with very mild impairment, indicating the need to anticipate that people well recovered from left‐hemisphere strokes will potentially develop long‐term fatigue. The trend towards more women reporting fatigue with comorbid mood problems and more men being in the non‐anxious fatigue category highlights the different fatigue profiles in the two genders and aids gender‐specific prediction of long‐term fatigue. While larger cohorts with a full spectrum of affective symptoms commonly comorbid with post‐stroke fatigue are necessary to identify a fuller range of clusters, the results of this study suggest any fatigue intervention study design must identify the effects of intervention in each individual cluster, as indicated by this study, to avoid missing cluster‐specific effects, which may otherwise be obscured by a mixed cohort of target participants.

### Limitations

A noteworthy limitation of this study is the small number of subjects included in the clustering analysis. The use of a k‐means clustering method was chosen over other more sophisticated clustering methods, such as latent class clustering and Gaussian mixture models, due to the small number of datapoints, as k‐means is known to perform better with smaller datasets. Moreover, by carefully selecting the minimum number of variables with known associations with fatigue, the robustness of procedures was maintained. Several previous studies have shown significant associations with cognitive dysfunction [7, 36, 37, 38]. The focus of the present paper was mainly physical variables, and inclusion of cognitive measures could result in more clusters. This exclusion of cognitive measures could be seen as a limitation.

## CONCLUSION

In conclusion, clustering revealed distinct subcategories of high post‐stroke fatigue defined by side of stroke, impairment and mood. Such clustering of high fatigue is essential to understand how fatigue emerges in a given population and is critical for developing effective interventions. The demonstration of the capability of clustering methods to inform phenotyping of post‐stroke fatigue is a significant step forward and represents a major contribution made by this paper. Building on these foundations, future investigations must focus on definitively establishing phenotypes of post‐stroke fatigue in larger samples with carefully selected structural, physiological, behavioural and self‐reported variables associated with post‐stroke fatigue.

## AUTHOR CONTRIBUTIONS

**Annapoorna Kuppuswamy:** Formal analysis; conceptualization; funding acquisition; writing – original draft; writing – review and editing. **William De Doncker:** Data curation.

## FUNDING INFORMATION

This work was supported by the Wellcome Trust (202346/Z/16/Z).

## CONFLICT OF INTEREST STATEMENT

The authors report no conflict of interest.

## Data Availability

The data that support the findings of this study are available from the corresponding author upon reasonable request.

## References

[1] Egerton T , Hokstad A , Askim T , Bernhardt J , Indredavik B . Prevalence of fatigue in patients 3 months after stroke and association with early motor activity: a prospective study comparing stroke patients with a matched general population cohort. BMC Neurol. 2015;15:181. doi:10.1186/s12883-015-0438-6 26444541 PMC4596493

[2] Feigin VL , Barker‐Collo S , Parag V , et al. Prevalence and predictors of 6‐month fatigue in patients with ischemic stroke: a population‐based stroke incidence study in Auckland, New Zealand, 2002‐2003. Stroke. 2012;43(10):2604‐2609. doi:10.1161/STROKEAHA.112.660886 22851549

[3] Cumming TB , Packer M , Kramer SF , English C . The prevalence of fatigue after stroke: a systematic review and meta‐analysis. Int J Stroke. 2016;11(9):968‐977. doi:10.1177/1747493016669861 27703065

[4] De Doncker W , Dantzer R , Ormstad H , Kuppuswamy A . Mechanisms of poststroke fatigue. J Neurol Neurosurg Psychiatry. 2018;89(3):287‐293. doi:10.1136/jnnp-2017-316007 28939684

[5] Cumming TB , Yeo AB , Marquez J , et al. Investigating post‐stroke fatigue: an individual participant data meta‐analysis. J Psychosom Res. 2018;113:107‐112. doi:10.1016/j.jpsychores.2018.08.006 30190042

[6] Naess H , Lunde L , Brogger J , Waje‐Andreassen U . Fatigue among stroke patients on long‐term follow‐up. The Bergen stroke study. J Neurol Sci. 2012;312(1–2):138‐141. doi:10.1016/j.jns.2011.08.002 21862037

[7] Radman N , Staub F , Aboulafia‐Brakha T , Berney A , Bogousslavsky J , Annoni JM . Poststroke fatigue following minor infarcts: a prospective study. Neurology. 2012;79(14):1422‐1427. doi:10.1212/WNL.0b013e31826d5f3a 22955128

[8] Kuppuswamy A , Clark EV , Sandhu KS , Rothwell JC , Ward NS . Post‐stroke fatigue: a problem of altered corticomotor control? J Neurol Neurosurg Psychiatry. 2015;86(8):902‐904. doi:10.1136/jnnp-2015-310431 25886778

[9] Kjeverud A , Østlie K , Schanke AK , Gay C , Thoresen M , Lerdal A . Trajectories of fatigue among stroke patients from the acute phase to 18 months post‐injury: a latent class analysis. PLoS One. 2020;15(4):e0231709. doi:10.1371/journal.pone.0231709 32294142 PMC7159233

[10] Lerdal A , Bakken LN , Rasmussen EF , et al. Physical impairment, depressive symptoms and pre‐stroke fatigue are related to fatigue in the acute phase after stroke. Disabil Rehabil. 2011;33(4):334‐342. doi:10.3109/09638288.2010.490867 20521900

[11] Wang J , Gu M , Xiao L , et al. Association of Lesion Location and Fatigue Symptoms after Ischemic Stroke: a VLSM study. Front Aging Neurosci. 2022;14:902604. doi:10.3389/fnagi.2022.902604 35847675 PMC9277067

[12] Schaechter J , Kim M , Hightower B , Ragas T , Loggia M . Disruptions in structural and functional connectivity relate to poststroke fatigue. Brain Connect. 2022;13:15‐27. doi:10.1089/brain.2022.0021 35570655 PMC9942175

[13] Tang WK , Chen YK , Liang HJ , et al. Subcortical white matter infarcts predict 1‐year outcome of fatigue in stroke. BMC Neurol. 2014;14:234. doi:10.1186/s12883-014-0234-8 25496671 PMC4272810

[14] Visser MM , Maréchal B , Goodin P , et al. Predicting modafinil‐treatment response in poststroke fatigue using brain morphometry and functional connectivity. Stroke. 2019;50(3):602‐609. doi:10.1161/STROKEAHA.118.023813 30777001

[15] Kuppuswamy A , Clark EV , Turner IF , Rothwell JC , Ward NS . Post‐stroke fatigue: a deficit in corticomotor excitability? Brain. 2015;138(Pt 1):136‐148. doi:10.1093/brain/awu306 25367024 PMC4441078

[16] Ondobaka S , De Doncker W , Ward N , Kuppuswamy A . Neural effective connectivity explains subjective fatigue in stroke. Brain. 2021;145:285‐294. doi:10.1093/brain/awab287 PMC8967104 34791073

[17] Mitchell AJ , Sheth B , Gill J , et al. Prevalence and predictors of post‐stroke mood disorders: a meta‐analysis and meta‐regression of depression, anxiety and adjustment disorder. Gen Hosp Psychiatry. 2017;47:48‐60. doi:10.1016/j.genhosppsych.2017.04.001 28807138

[18] Johansson S , Kottorp A , Lee KA , Gay CL , Lerdal A . Can the fatigue severity scale 7‐item version be used across different patient populations as a generic fatigue measure–a comparative study using a Rasch model approach. Health Qual Life Outcomes. 2014;12:24. doi:10.1186/1477-7525-12-24 24559076 PMC3936846

[19] Scheerer NE , Curcin K , Stojanoski B , et al. Exploring sensory phenotypes in autism spectrum disorder. Mol Autism. 2021;12(1):67. doi:10.1186/s13229-021-00471-5 34641960 PMC8507349

[20] Clementz BA , Sweeney JA , Hamm JP , et al. Identification of distinct psychosis biotypes using brain‐based biomarkers. Am J Psychiatry. 2016;173(4):373‐384. doi:10.1176/appi.ajp.2015.14091200 26651391 PMC5314432

[21] English CK , Simpson DB , Billinger S , et al. EXPRESS: a roadmap for research in post‐stroke fatigue: consensus‐based core recommendations from the third stroke recovery and rehabilitation roundtable. Int J Stroke. 2023;17474930231189135. doi:10.1177/17474930231189135 PMC10811972 37424273

[22] Schepers VP , Visser‐Meily AM , Ketelaar M , Lindeman E . Poststroke fatigue: course and its relation to personal and stroke‐related factors. Arch Phys Med Rehabil. 2006;87(2):184‐188. doi:10.1016/j.apmr.2005.10.005 16442970

[23] Winward C , Sackley C , Metha Z , Rothwell PM . A population‐based study of the prevalence of fatigue after transient ischemic attack and minor stroke. Stroke. 2009;40(3):757‐761. doi:10.1161/STROKEAHA.108.527101 19131658

[24] Wassermann EM . Variation in the response to transcranial magnetic brain stimulation in the general population. Clin Neurophysiol. 2002;113(7):1165‐1171. 12088713 10.1016/s1388-2457(02)00144-x

[25] Davidson T , Tremblay F . Hemispheric differences in corticospinal excitability and in transcallosal inhibition in relation to degree of handedness. PLoS One. 2013;8(7):e70286. doi:10.1371/journal.pone.0070286 23936180 PMC3723808

[26] Hammond G . Correlates of human handedness in primary motor cortex: a review and hypothesis. Neurosci Biobehav Rev. 2002;26(3):285‐292. doi:10.1016/S0149-7634(02)00003-9 12034131

[27] Klein PA , Duque J , Labruna L , Ivry RB . Comparison of the two cerebral hemispheres in inhibitory processes operative during movement preparation. Neuroimage. 2016;125:220‐232. doi:10.1016/j.neuroimage.2015.10.007 26458519 PMC4699690

[28] Verstynen T , Ivry RB . Network dynamics mediating ipsilateral motor cortex activity during unimanual actions. J Cogn Neurosci. 2011;23(9):2468‐2480. doi:10.1162/jocn.2011.21612 21268666

[29] Hervé PY , Leonard G , Perron M , et al. Handedness, motor skills and maturation of the corticospinal tract in the adolescent brain. Hum Brain Mapp. 2009;30(10):3151‐3162. doi:10.1002/hbm.20734 19235881 PMC6870659

[30] Ziemann U , Hallett M . Hemispheric asymmetry of ipsilateral motor cortex activation during unimanual motor tasks: further evidence for motor dominance. Clin Neurophysiol. 2001;112(1):107‐113. doi:10.1016/s1388-2457(00)00502-2 11137667

[31] Corbetta M , Shulman GL . Control of goal‐directed and stimulus‐driven attention in the brain. Nat Rev Neurosci. 2002;3(3):201‐215. doi:10.1038/nrn755 11994752

[32] Mevorach C , Humphreys GW , Shalev L . Opposite biases in salience‐based selection for the left and right posterior parietal cortex. Nat Neurosci. 2006;9(6):740‐742. doi:10.1038/nn1709 16699505

[33] Giovannelli F , Borgheresi A , Balestrieri F , et al. Modulation of interhemispheric inhibition by volitional motor activity: an ipsilateral silent period study. J Physiol (Lond). 2009;587(Pt 22):5393‐5410. doi:10.1113/jphysiol.2009.175885 19770195 PMC2793872

[34] Ondobaka S , Ward N , Kuppuswamy A . Inter‐hemispheric inhibition in stroke survivors is related to fatigue and cortical excitability. *bioRxiv*. 2019:831511. doi:10.1101/831511

[35] De Doncker W , Ondobaka S , Kuppuswamy A . Effect of transcranial direct current stimulation on post‐stroke fatigue. J Neurol. 2021;268:2831‐2842. doi:10.1007/s00415-021-10442-8 33598767 PMC8289762

[36] Ulrichsen KM , Alnaes D , Kolskår KK , et al. Dissecting the cognitive phenotype of post‐stroke fatigue using computerized assessment and computational modeling of sustained attention. Eur J Neurosci. 2020;52(7):3828‐3845. doi:10.1111/ejn.14861 32530498

[37] Kuppuswamy A , Harris AM , Doncker WD , Alexander A , Lavie N . Diminished distractor filtering with increased perceptual load and sustained effort explains attention deficit in post‐stroke fatigue. *bioRxiv*. 2022:2022.03.17.484709. doi:10.1101/2022.03.17.484709

[38] Pihlaja R , Uimonen J , Mustanoja S , Tatlisumak T , Poutiainen E . Post‐stroke fatigue is associated with impaired processing speed and memory functions in first‐ever stroke patients. J Psychosom Res. 2014;77:380‐384. doi:10.1016/j.jpsychores.2014.08.011 25218164

